# Quantitative survey of multiple CpGs from 5 genes identifies CpG methylation panel discriminating between high- and low-grade cervical intraepithelial neoplasia

**DOI:** 10.1186/s13148-014-0037-1

**Published:** 2015-01-22

**Authors:** Xiaoyi Tian, Di Chen, Ran Zhang, Jun Zhou, Xiaozhong Peng, Xiaolin Yang, Xiuru Zhang, Zhi Zheng

**Affiliations:** Department of Biochemistry and Molecular Biology, Institute of Basic Medical Sciences, Chinese Academy of Medical Sciences and School of Basic Medicine, Peking Union Medical College, No. 5 Dong Dan San Tiao, Beijing, 100005 China; Department of Pathology, Aerospace Central Hospital, No. 15 Yuquan Road, Beijing, 100049 China; Department of Biomedical Engineering, Institute of Basic Medical Sciences, Chinese Academy of Medical Sciences and School of Basic Medicine, Peking Union Medical College, No. 5 Dong Dan San Tiao, Beijing, 100005 China; Department of Pathology, Beijing Tiantan Hospital Affiliated with Capital Medical University, No. 6 Tiantan Xili, Beijing, 100050 China

**Keywords:** Cervical cancer, Cervical intraepithelial neoplasia (CIN), DNA methylation, High-grade CIN, Biomarkers

## Abstract

**Background:**

Studies of methylation biomarkers for cervical cancer often involved only few randomly selected CpGs per candidate gene analyzed by methylation-specific PCR-based methods, with often inconsistent results from different laboratories. We evaluated the role of different CpGs from multiple genes as methylation biomarkers for high-grade cervical intraepithelial neoplasia (CIN).

**Results:**

We applied a mass spectrometry-based platform to survey the quantitative methylation levels of 34 CpG units from *SOX1*, *PAX1*, *NKX6-1*, *LMX1A*, and *ONECUT1* genes in 100 cervical formalin-fixed paraffin-embedded (FFPE) tissues. We then used nonparametric statistics and Random Forest algorithm to rank significant CpG methylations and support vector machine with 10-fold cross validation and 200 times bootstrap resampling to build a predictive model separating CIN II/III from CIN I/normal subjects. We found only select CpG units showed significant differences in methylation between CIN II/III and CIN I/normal groups, while mean methylation levels per gene were similar between the two groups for each gene except *PAX1*. An optimal classification model involving five CpG units from *SOX1*, *PAX1*, *NKX6-1*, and *LMX1A* achieved 81.2% specificity, 80.4% sensitivity, and 80.8% accuracy.

**Conclusions:**

Our study suggested that during CIN development, the methylation of CpGs within CpG islands is not uniform, with varying degrees of significance as biomarkers. Our study emphasizes the importance of not only methylated marker genes but also specific CpGs for identifying high-grade CINs. The 5-CpG classification model provides a promising biomarker panel for the early detection of cervical cancer.

**Electronic supplementary material:**

The online version of this article (doi:10.1186/s13148-014-0037-1) contains supplementary material, which is available to authorized users.

## Background

Cervical cancer is one of the leading causes of cancer-related mortality in women worldwide. Cervical intraepithelial neoplasia (CIN) is a premalignant transformation and abnormal growth (dysplasia) of squamous cells of the cervix. Early screening together with timely treatment of precancerous lesions can substantially improve clinical outcome, thus offering a unique opportunity to cervical cancer management. The widely used screening strategy, cytology-based Pap smear, has been associated with a significant reduction of cancer incidence rate and mortality [1].

Besides finding cervical carcinomas, cervical cancer screening aims to identify high-grade intraepithelial lesions (corresponding to histological grades CIN II and CIN III) which require surgical procedures to prevent further progression. Low-grade intraepithelial lesions (corresponding to histological grade CIN I), on the other hand, should not be over-treated for such procedures as they have high potential to spontaneously regress to normal [2]. However, the sensitivity of Pap smears for the detection of CIN II or higher grades is generally low [3,4]. On the other hand, the highly sensitive diagnostic high-risk human papillomavirus (HPV) DNA testing tends to give false positives [5-9]. A third strategy is direct colposcopy [10], which requires interpretational expertise, is not amenable to high throughput processing, and has low positive predictive values for low-grade squamous intraepithelial lesions [4,11]. Finally, even for histopathology specimen of cervical biopsies, objective CIN diagnosis can be sometimes challenging. The reproducibility of cervical histopathologic interpretations was moderate and equivalent to the reproducibility of monolayer cytologic interpretations [12]. Thus, an objective, high-throughput approach with high sensitivity and specificity is urgently needed for early diagnosis of cervical cancer.

Numerous investigations have reported that gene-specific hypermethylation occurring in pre-invasive and invasive phase of cervical cancer may be promising biomarkers for early diagnosis [13-21]. A review of the results of 51 published cervical cancer methylation studies involving 68 different genes concluded, however, that no single methylation marker from these studies was suitable as a cervical cancer biomarker [13]. Most identified biomarkers, with a few exception [22,23], lacked sufficient independent validations. Currently, therefore, it is as important to validate existing candidates as to identify additional ones. Another concern regarded inconsistent results in methylation studies. Most of these studies used methylation-specific PCR (MSP) or quantitative methylation-specific PCR (QMSP) methods [24], analyzing in each gene one or two CpGs which were selected randomly as those feasible for primer/probe design, assuming hypermethylation is uniform across CpG promoter and the analyzed CpGs are representative. The measured methylation frequencies varied widely for the same gene even between studies that used common specimen or similar assays [13]. A recent study by Lai et al. [18], on a Chinese cohort of squamous cell carcinoma (SCC), identified six novel genes (*SOX1*, *PAX1*, *NKX6-1*, *LMX1A*, *ONECUT1* and *WT1*) as more frequently methylated in SCC tissues than in normal controls. Some of the markers were verified by the same laboratory using QMSP (MethyLight) [25,26]. However, two of these methylation markers had different performance between the two studies [18,25]. Moreover, another study by an independent laboratory using QMSP on liquid-based cytology samples from a UK cohort found that only one of these genes, *SOX1*, was able to discriminate between high-grade squamous intraepithelial lesions and controls [15]. Surprisingly, although such disturbing discrepancies cast considerable doubts on the validity of identified biomarker candidates, little study was undertaken to examine their potential causes.

We suspected that different CpGs assayed by different groups for the same genes may be a major factor contributing to the result variances, and decided to systematically evaluate different CpGs from multiple genes as methylation biomarkers. For early detection of cervical cancer, it is clinically more useful to find epigenetic correlates discriminating between histologically distinct CINs than between SCC and normal cervices, yet few studies have focused exclusively on CIN development. We set to evaluate the utility of methylation biomarkers in distinguishing high-grade from low-grade CIN lesions. Our aims were therefore twofold: (1) to evaluate the relative importance of different CpGs as methylation biomarkers and thus decide whether randomly selecting CpGs to assay, as practiced in most methylation biomarker studies, is justified and (2) to find an optimal panel of candidate hypermethylated CpGs with high sensitivity and specificity for precancerous CIN II or CIN III.

To these ends, we evaluated 34 CpG units from five candidate genes, using definitively diagnosed FFPE tissue specimens from an independent cohort of 100 Chinese precancerous cervical patients and normal controls, who shared a common genetic background with the subjects of the original gene-discovery study [18]. We used a matrix-assisted laser desorption ionization time-of-flight mass spectrometry (MALDI-TOF MS)-based DNA methylation quantification technology (EpiTYPER, Sequenom) [27], which is fundamentally different from the commonly used MSP or QMSP methods. Our method yields direct quantification of the percentage of DNA methylated in a CpG unit, with results highly concordant with bisulfite sequencing [28]. This technology has already been applied to evaluate methylation patterns of leukemia [29] and non-small cell lung cancer [30].

To rank CpG units with discriminating power, we used traditional nonparametric statistics as well as Random Forest, a method particularly well suited for analyzing mass spectrometry data in studies of biomarker identification for cancer classification [31]. We then used support vector machine (SVM) [32-35] with cross-validation and bootstrap resampling, which randomly partitioned the tested samples into training and validation sets, to construct an inferred model and assessed the predicative power of the model. Our results showed that choosing the right CpG unit to assay is critical, and a panel of multiple specific CpG methylation constructed by computerized algorithm allowed us to separate high-grade CIN from low-grade or healthy subjects with high accuracy, providing a candidate biomarker panel for early detection of cervical cancer development.

## Results

### Survey of CpG methylation of five genes by MALDI-TOF-based EpiTYPER assay

**Figure 1:**
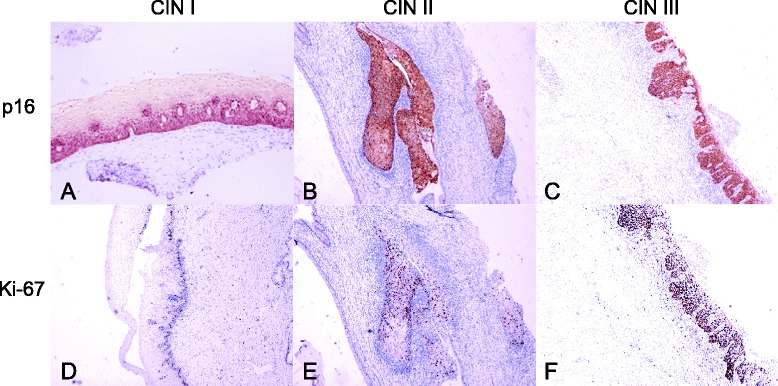
**CINs with p16 and Ki-67 immunostaining.** Immunohistochemical examination of p16 **(A–C)** and Ki-67 **(D–F)** protein expressions in histologically CIN I **(A, D)**, CIN II **(B, E)**, and CIN III **(C, F)** tissues. Magnifications × 40.

A total of 100 FFPE cervical samples with histopathological classifications of normal (*N* = 16), CIN I (*N* = 31), and CIN II or CIN III (*N* = 53 including 4 CIN II and 49 CIN III) were obtained retrospectively from a cohort of ethnic Han Chinese women. The CIN samples were all tested HPV positive (data not shown), and consensus histological diagnoses were provided independently by two pathologists, with confirmation by p16 and Ki-67 immunohistochemistry staining (Figure 1). There was no significant age difference between different groups (Table 1).

**Table 1:** **Sample characteristics and number of samples whose CpG islands for each gene were successfully amplified for EpiTYPER analysis**

**Stages (** ***N*** **)**	**Mean Age ± SD**	***LMX1A***	***SOX1***	***ONECUT1***	***NKX6.1***	***PAX1***
Normal (16)	48.3 ± 10.4	16	15	16	15	16
CIN I (31)	43.6 ± 11.2	23	24	31	30	22
CIN II + III(53)	41.0 ± 7.8	39	45	47	35	34
Total (100)		78	84	94	80	72

*PAX1*: paired box 1; *SOX1*: SRY (sex determining region Y)-box 1; *LMX1A*: LIM homeobox transcription factor 1, alpha; *NKX6-1*: NK6 homeobox 1; *ONECUT1*: one cut homeobox 1; CIN: cervical intraepithelial neoplasia; SD: standard deviation.

**Figure 2:**
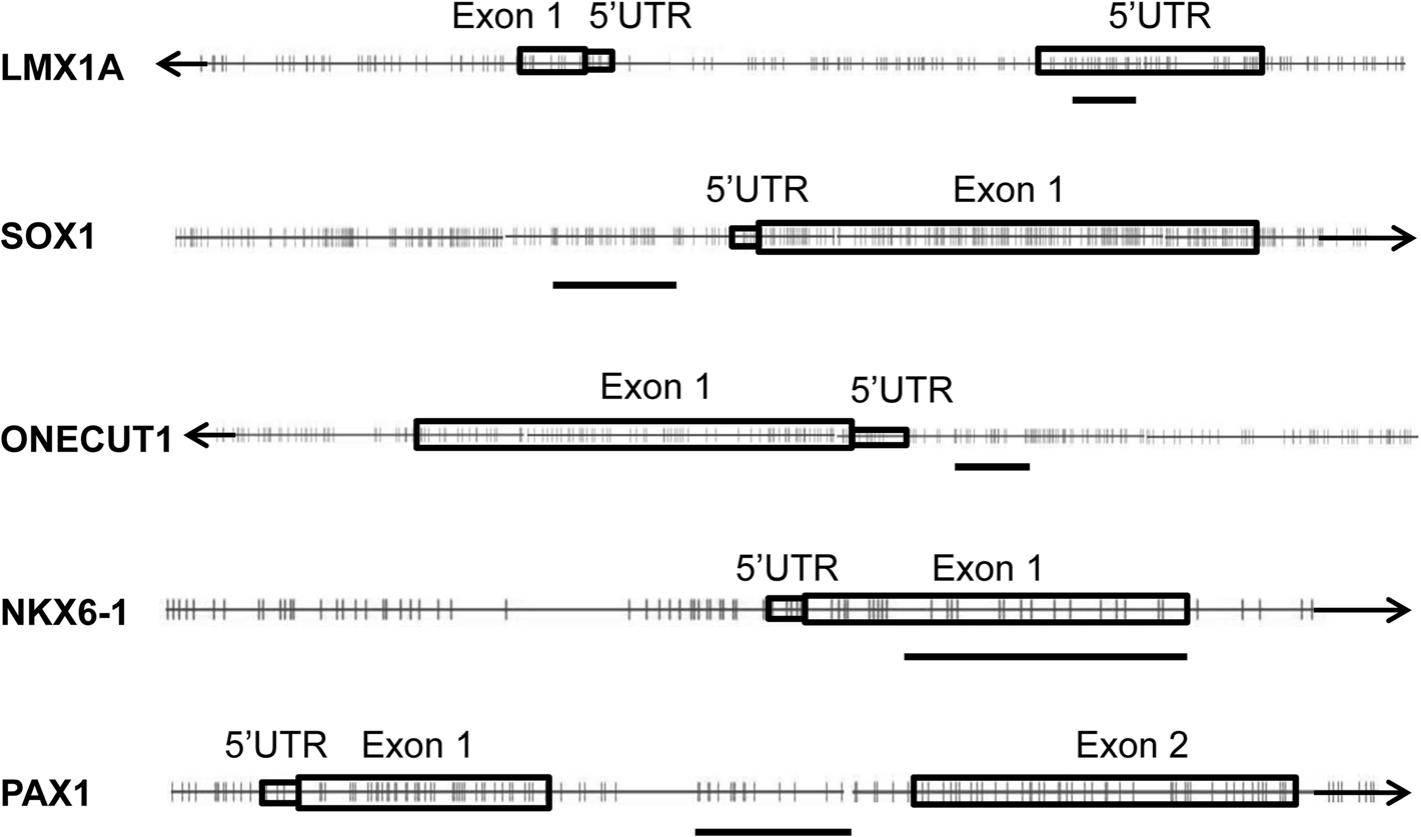
**The positions of CpGs analyzed by EpiTYPER.** Drawings are schematic and not to scale. The orientation of each gene is indicated by the *arrow* at the end. *Boxes* indicate exons or UTRs; *vertical lines* indicate individual CpGs in the CGI regions, and the *horizontal bars* indicate the regions analyzed by EpiTYPER.

To analyze the methylation status of *PAX1*, *NKX6-1*, *SOX1*, *LMX1A*, and *ONECUT1*, a CpG island (CGI) for each gene was chosen for amplification (Figure 2 and Table S1 in Additional file 1). Each CGI contained five to eight CpG units that can be analyzed by EpiTYPER in this study (Table 2).

**Table 2:** **The number of CpG units for each gene**

**Genes**	**Total CpG units**	**CpG units analyzed**
*LMX1A*	16	8
*SOX1*	18	7
*ONECUT1*	10	8
*NKX6.1*	8	6
*PAX1*	7	5
Total	59	34

A CpG unit harbors one or more CpGs in a unique enzymatic cleavage fragment analyzed by EpiTYPER. Only CpG units residing in cleaved mass fragments that were within MS analytical window (*m*/*z* 4000–10000) were analyzed.

Upon DNA extraction, bisulfite treatment, and PCR amplification, 72–94% of the samples, depending on the target gene of interest, generated sufficient amplicons that were amenable to subsequent EpiTYPER analysis (Table 1), suggesting that the assay design and sample processing protocol were suitable for the archival FFPE samples. The EpiTYPER is capable of simultaneously determining all applicable CpG units within a CGI amplicon in one well. Quantitative methylation assessment of a total of 34 CpG units (Table 2) in 100 individuals was completed in two 384-well plates in a single day.

**Figure 3:**
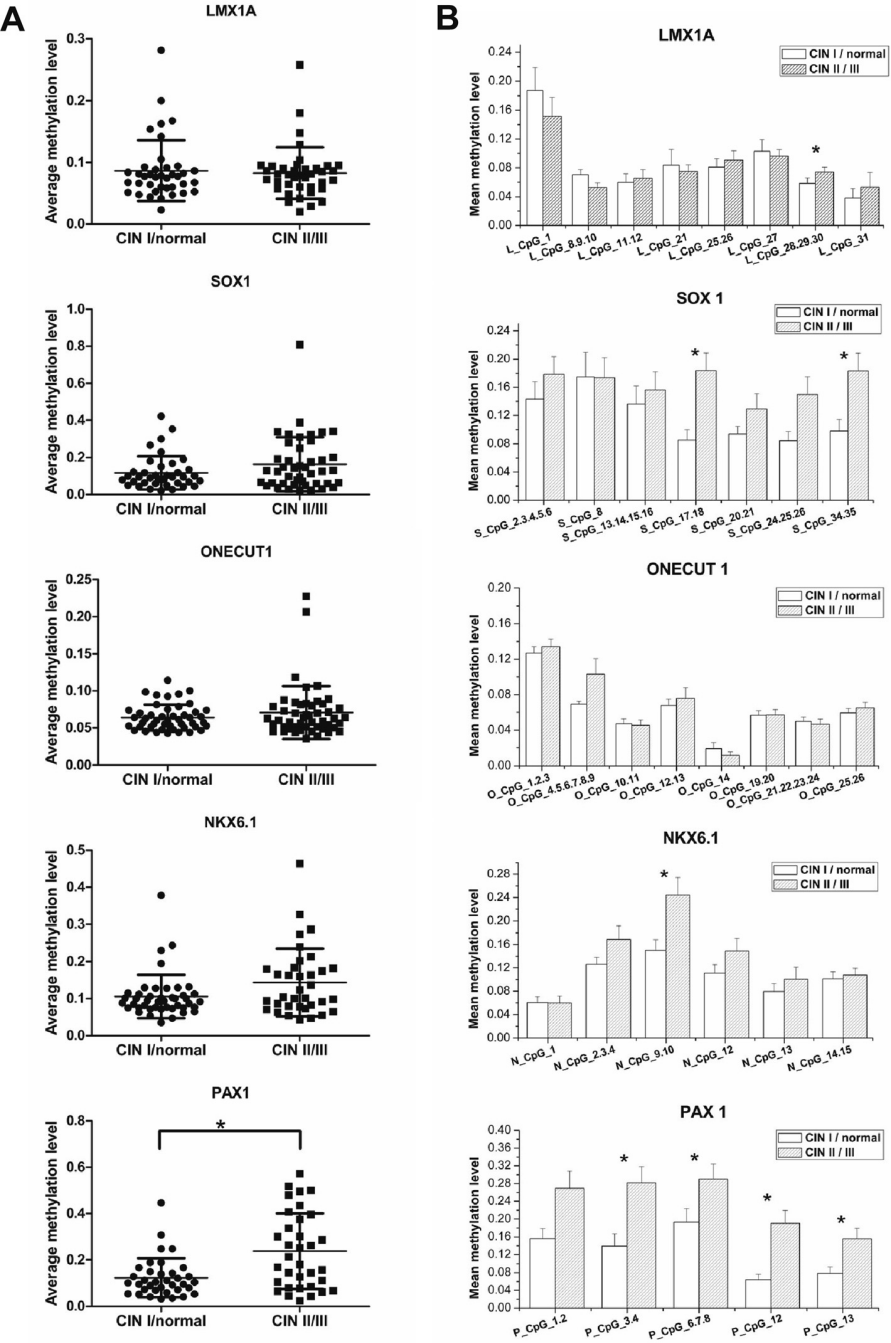
**The methylation patterns of the five genes. (A)** Dot plot of the methylation levels for each gene. Each *dot* represents a sample, with methylation level averaged over all CpG units analyzed for the gene. **(B)** Bar graph of the methylation levels of individual CpG units. *Open* and *filled columns* denote CIN I/normal and CIN II/III group, respectively. Each *bar* represents mean methylation of all samples in the group. Error bars indicate SEM; *P < 0.05 for Mann-Whitney *U* test.

When we examined CGIs of candidate genes, we observed unexpectedly that the mean methylation levels of the CGIs of four of the five genes were not statistically different between CIN II/III and CIN I/normal groups (Figure 3A), indicating that during CIN development, the overall methylation status of the examined CGIs of *NKX6-1*, *SOX1*, *LMX1A*, and *ONECUT1* did not change. However, when we examined individual CpG units, statistically significant difference in methylation between the CIN II/III and CIN I/normal groups emerged for eight CpG units (Figure 3B). *PAX1* had the highest methylation level among the five genes, with the methylation level of four CpG units being significantly different between the two groups (*P* < 0.05). Although the overall methylation level of *LMX1A* was low, one CpG unit (*L*_CpG 28.29.30) was differentially methylated between the two groups (Figure 3B). *NKX6-1* and *SOX1* exhibited a moderate methylation level and, respectively, contained one (*N*_CpG 9.10) and two (*S_* CpG 17.18 and *S_* CpG 34.35) significant CpG units. *ONECUT1* contained no significantly methylated CpG units (Figure 3B). These data suggested that during CIN development, the methylation within CGIs was not uniform.

### Validation by bisulfite sequencing

**Figure 4:**
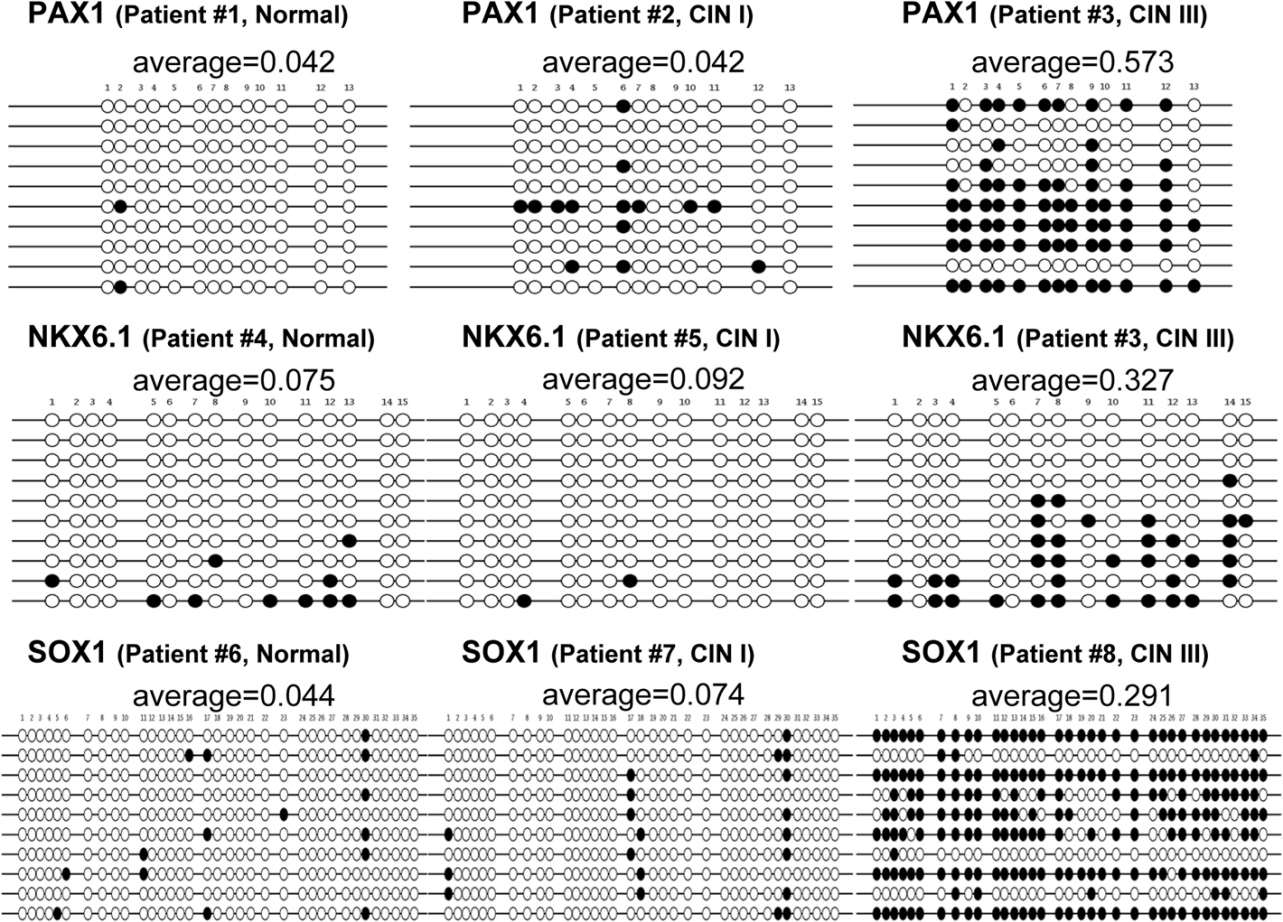
**Bisulfite sequencing (BS) of CpGs assayed by EpiTYPER.** Three genes were bisulfite-sequenced in eight cervical samples of various stages. In each panel, sample ID is shown at the *top*, and EpiTYPER results are shown *below* the gene name as the average level for all measured CpGs. BS results are summarized as *filled circles* representing methylated CpGs and *open circles* representing unmethylated CpGs. Each *line* is an independently sequenced clone. Each *column* is a CpG of the gene.

To validate the EpiTYPER methylation results using independent methods, we did bisulfite sequencing for three genes on eight samples (Figure 4). As expected, the sequencing results were in accordance with quantitative EpiTYPER results. Moreover, sequencing confirmed that the DNA methylation was not uniform, as some specific CpGs tended to exhibit more frequent methylation than other CpGs (Figure 4).

### Significance ranking of CpG units by Random Forest

**Figure 5:**
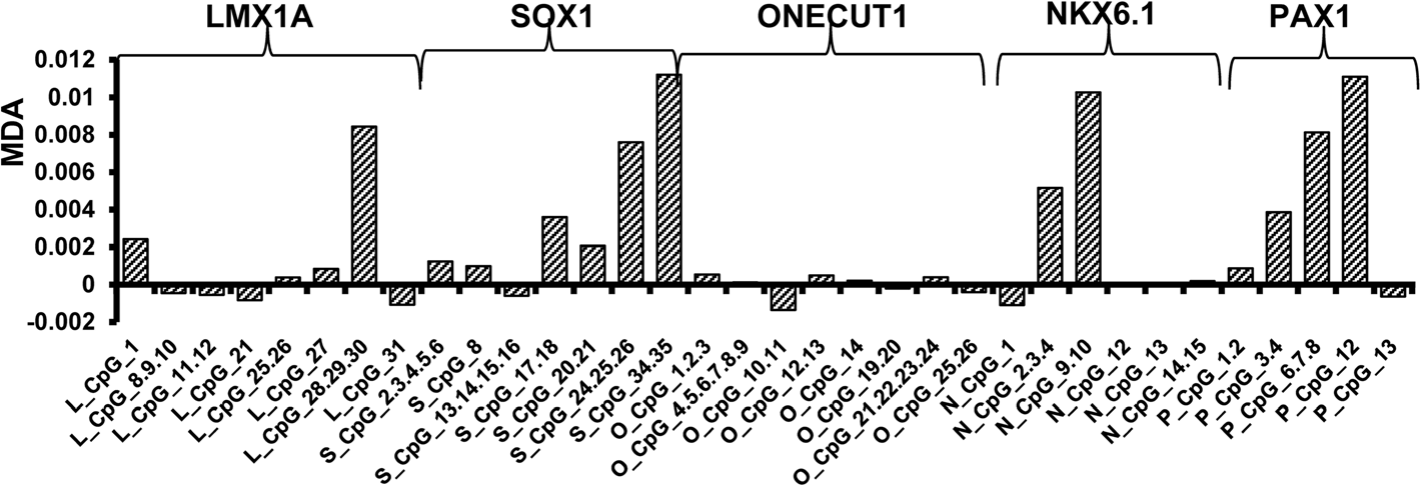
**Feature importance for prediction of CIN II/III according to Random Forest algorithm.** MDA: mean decrease in accuracy.

To evaluate the contribution of 34 CpG units to the separation of CIN II/III subjects from CIN I/normal ones, we employed the Random Forest algorithm (see “Methods”) in addition to the standard nonparametric statistical method; Figure 5 shows the mean decrease in accuracy (MDA) values of the 34 CpG units, with higher MDA indicating increasing importance of a CpG unit as predictor [36]. We tested the performance of classifiers constructed by the assembly of different features iteratively. When the selected features were *PAX1_CpG12*, *SOX1_CpG34.35*, *LMX1A_ CpG28.29.30*, *NKX6-1_CpG9.10*, and *PAX1_CpG6.7.8*, the classifiers achieved the optimal performance. Table 3 presents the Mann-Whitney *U* test result of these five CpG units.

**Table 3:** **Summary of nonparametric statistics for the selected CpG units in building classifier**

**CpG unit**	**Methylation level (mean ± s.e)**		***P*** **value**
	**Normal/CIN I**	**CIN II/III**	
P_CpG_6.7.8	0.193 ± 0.0308	0.290 ± 0.0344	0.0383
P_CpG_12	0.064 ± 0.0123	0.191 ± 0.0293	0.0097
L_CpG_28.29.30	0.059 ± 0.0076	0.074 ± 0.0067	0.0271
N_CpG_9.10	0.150 ± 0.0182	0.244 ± 0.0304	0.0003
S_CpG_34.35	0.098 ± 0.01615	0.183 ± 0.0257	0.029

### Classification model built by SVM

To build optimal classification model, the methylation levels of the above five CpG units were entered into a SVM with radial basis function (RBF) kernel (see “Methods”). When *C* and *γ* were 410 and 14, the classifiers showed the optimal and robust performance. With 200 times bootstrap resampling and 10-fold cross validation, the classification model showed high predictive power with sensitivity, specificity, and accuracy of 0.804 ± 0.028, 0.812 ± 0.008, and 0.808 ± 0.014 (mean ± SD), respectively (Table 4).

**Table 4:** **Evaluation parameters of SVM classifier model trained by the best parameter setting**

**Measures**	**Mean**	**SD**
Sensitivity	0.804	0.028
Specificity	0.812	0.008
Accuracy	0.808	0.014

SD: standard deviation.

## Discussion

CpG hypermethylation of key genes involved in cervical cancer development may be promising biomarkers for early diagnosis [13-18,23]. However, progress in the field of cervical methylation biomarker discovery has been hampered by inconsistent results that defy validations [13]. Most of the previous studies used nonquantitative MSPs [18,37] that are highly sensitive but with the drawback of being unable to distinguish tumors with substantial methylation from those with biologically insignificant methylation levels [28]. Recently developed quantitative MSP (QMSP) such as MethyLight provides a better alternative and is becoming increasingly used in methylation studies [24,38,39]. However, QMSP will only detect methylation of few CpGs (equivalent of one CpG unit in our assay, usually 2 ~ 3 CpGs each) [24]. It is difficult to design assays in CpG-rich areas without having the probe overlapping flanking CpGs, and with the probe having sufficient annealing temperature to achieve robust annealing and specificity in the highly AT-rich sequence after bisulfite conversion. This makes QMSP limited in applications and perhaps explains the sometimes variable results obtained for the same gene using different primer/probe designs [15,18,25]. In contrast, primers used in EpiTYPER assay did not involve CpGs (Table S1 in Additional file 1), resulting in more consistent results. The EpiTYPER assay showed much better correlation with the gold-standard sequencing results than the MSP-based assays [28]. The technique could analyze almost all CpGs covered within one amplicon for a gene, instead of 2 ~ 3 CpGs randomly chosen by QMSP. We used this novel quantitative platform to survey 5 ~ 8 CpG units (containing 13 ~ 35 CpGs) within a CGI of each gene for five genes. We found that randomly selecting CpGs to assay gene methylation can be problematic, as CpG methylation is not uniform during CIN development (Figure 3B). Of all genes except *PAX1*, the overall methylation status, defined as the averaged level of all CpGs within the CGI, was similar between CIN II/III and CIN I/normal(Figure 3A). This is in contrast to the conclusion based on a much limited number of CpGs using MSP [18,25]. Only select CpG units may be used as markers distinguishing CIN II/III from CIN I/normal samples (Figure 3B). Consistent with our observation, other reports has demonstrated that aberrant DNA methylation of only specific CpGs within the CGI are responsible for the downregulation of gene expression [40-44], and more recently, a substantial number of studies reported a specific, single CpG can function as strong prognostic or predictive indicators in various cancers [28,45-47].

Our findings highlight the importance of studying the detailed methylation pattern within a CGI, as they reveal the temporal complexity of DNA methylation during cervical cancer development, and emphasize the importance of not only methylated marker genes but also specific CpGs for identifying high-grade CINs. Therefore, choosing the right CpG unit to assay is critical, and previous inconsistencies among different labs regarding methylation status of the same genes may be due to CpG choices.

We also note that significant CpG units for CIN development can reside beyond the promoter, in exonic or intronic regions as well (Figure 2), just as CpG methylation outside of promoter region can be responsible for tumor suppressor inactivation in breast cancer [48]. Although we did not evaluate all CpGs of these marker genes, our original findings could provide diagnostically useful methylation biomarkers for high-grade CIN. Moreover, MALDI-TOF-based technology gave consistent results to assay these CpG markers in a multiplexed, high-throughput fashion suitable for clinical applications.

Diagnostic classifiers built on multigene methylation panels have shown better performance in predicting a wide variety of tumors [49]. However, such studies commonly associated with the overfitting problem [50,51]. To overcome this, we used SVM to construct the classifier model [52-54] and coupled with a procedure of 10-fold cross validation (in which our samples were partitioned into randomly assigned training and testing sets for the model to be validated 10 times) and 200 times bootstrap resampling (in which the partitioning and cross-validation was randomized and repeated 200 times). Such procedures help reduce overfitting and provide a reliable estimate of the performance of the model [55]. Compared with classification methods used in previous studies [18,25], SVM is a statistical learning method with greater accuracy in diagnostic ability [32,33,54,56] and with more consistent performance at our sample size [57].

Hypermethylated genes selected to predict invasive cervical cancer achieved a sensitivity about 90% according to previous study. However, the high-grade CIN lesions were predicted with much lower sensitivity (~70%) [20,58]. Our panel of CpG units obtained a high sensitivity and specificity of ~80%, achieving a valuable balance between sensitivity and specificity in identifying high-risk samples. The high specificity of our classifier would be particularly suitable for developing countries like China, where cervical cancer prevalence remained relatively high.

Identification of a set of reliable CIN biomarkers serves as a foundation for potential future applications such as quality assurance of histopathology classifications and noninvasive cervical cancer screening if these markers are validated in exfoliated cell samples from cervical scrapings or Pap smears. Our panel of CpG units and the EpiTYPER platform can potentially be a part of an objective, high-throughput strategy for early cervical cancer detection.

## Conclusions

Our findings highlight the significance of studying the detailed methylation pattern within a CGI and emphasize the importance of not only methylated marker genes but also specific CpGs for identifying high-grade CINs. We demonstrated the value of the MALDI-TOF technology in methylation biomarker identification and obtained a five-CpG panel with a promising potential as a biomarker for the early detection of cervical cancer.

## Methods

### Samples

Formalin-fixed paraffin-embedded (FFPE) cervical biopsy samples were obtained from outpatients visiting the Beijing Aerospace Central Hospital from 2007 to 2012. All histological specimens were tested for HPV DNA (Hybrid Capture-2 kit; Qiagen, Gaithersburg, MD) and for p16 and Ki-67 immunostaining (Beijing Zhong Shan Golden Bridge Biological Technology Co., Ltd.) [59]. The specimens were reviewed independently by two expert pathologists from the Departments of Pathology at Aerospace Central Hospital and Beijing Tiantan Hospital, and only concordant, clearly unambiguous specimens were chosen for the study. Exclusion criteria included uncertain histopathological classification, pregnancy, chronic or acute systemic viral infections, presence of other cancers, skin or genital warts, and an immunocompromised state. Informed consents were obtained from all patients and controls. The study followed the ethical guidelines of the Institutional Review Board of the Aerospace Central Hospital.

### DNA preparation and bisulfite treatment

Genomic DNA was extracted from archival FFPE blocks using an established protocol [60]. DNA was quantified using the NanoDrop 2000 Spectrophotometer (Thermo Fisher Scientific Inc, CA.). Only DNA samples exhibiting an A260/A280 ratio between 1.8 and 2.0 were considered for further testing.

EZ DNA Methylation Kit (Zymo Research Corporation, CA) was used to modify extracted genomic DNA according to the manufacturer’s protocol with Sequenom recommendations.

### MALDI-TOF-MS-based DNA methylation analysis

MALDI-TOF-MS-based DNA methylation assay (EpiTYPER) was performed according to the manufacturer’s specification [27] (Sequenom Inc. CA). Bisulfite-modified genomic DNA was used as PCR template. Primers for PCR (Table S1 in Additional file 1), which do not contain CpGs and amplify both methylated and unmethylated sequences equally, were designed using EpiDesigner (http://www.epidesigner.com/) with the following constraints: (1) the amplicon was located in a CGI of the target gene, (2) the amplicon size is below 300 bp to increase the amplification success rate of FFPE samples, and (3) the amplicon covers as many CpGs as possible. The reverse primers included at the 5′ end a T7 promoter tag [5′-cagtaatacgactcactataggg-3′]. Only samples successfully amplified with a clear and specific PCR band at the expected size were included for further analysis. After PCR amplification, T7 RNA polymerase (Sequenom Inc.) was used to *in vitro* transcribe single-stranded RNA, which was then cleaved base-specifically by RNase A [27] (MassCLEAVE, Sequenom Inc.). The cleavage products, which contained either individual CpG or short stretches of adjacent CpGs, were analyzed using a MALDI-TOF mass spectrometer (Sequenom Inc). The peak areas of the mass signals derived from methylated and non-methylated template DNA were used to estimate the relative methylation level (valued from 0 to 1 or 0 to 100%). Methylation level for each CpG unit represents average of CpGs within the unit.

We used 100 and 0% methylated human DNA (EpiTect Control DNA Set, QIAGEN Inc. CA) as positive and negative controls, respectively, for the amplification and methylation determination. No-template controls were included for each amplicon to monitor PCR specificity.

### Bisulfite sequencing

We cloned the EpiTYPER PCR products into pGEM-T Easy vectors (Promega, WI). For each sample, Sanger sequencing was performed on 10 random individual clones using the 3730 automatic sequencer (Applied Biosystems, CA). Sequencing results were analyzed using the QUMA online software suite (http://quma.cdb.riken.jp/).

### Statistical analysis

For all statistical analysis in this study, the normal and CIN I samples were grouped into one category, so that all samples were classified as either CIN II/III or CIN I/normal. The relative methylation of each CpG unit in the dataset was analyzed as continuous variables. Nonparametric statistical analysis was performed with the two-tailed Mann-Whitney *U* test for unpaired comparisons (GraphPad Prism 5.01), with statistical significance set at *P* value <0.05.

Additionally, the significance of CpG unit was assessed using the MDA calculated by the feature selection algorithm of Random Forest [36] (https://code.google.com/p/randomforest-matlab). Two main parameters of Random Forest, *n*tree (the number of trees in the forest) and *m*try (the number of variables randomly chosen at each split in a tree), were set to 5000 and 6, respectively.

We used SVM with a RBF kernel for the classifiers. The SVM parameters (penalty parameter *C*, kernel parameter *γ*) were optimized using grid-search method [50]. Besides SVM, we used 10-fold cross-validation combined with 200 times bootstrapping sampling in constructing and evaluating the classification model. Thus, the original samples were randomly partitioned into 10 equal-sized subsets, 9 of which were used as training data and the remaining set for validation testing. The process was repeated 10 times to ensure each subset was used exactly once as the testing set. The random partition and cross-validation repeat 200 times altogether. The classification performances were assessed using the sensitivity, the specificity, and the accuracy of the classification [61]. All computational experiments were carried out in the MATLAB (Version 8.1) programming environment.

## Additional file

Additional file 1: Table S1. Primers, respective target sizes, and locations of the CGIs analyzed by EpiTYPER. L indicates left primer; R indicates right primer.

## Abbreviations

CIN: Cervical intraepithelial neoplasia
MSP: Methylation-specific PCR
HPV: Human papillomavirus
QMSP: Quantitative methylation-specific PCR
SCC: Squamous cell carcinoma
MALDI-TOF MS: Matrix-assisted laser desorption ionization time-of-flight mass spectrometry
CGI: CpG island
SVM: Support vector machine
FFPE: Formalin-fixed paraffin-embedded
MDA: Mean decrease in accuracy
RBF: Radial basis function

## References

[1] Gustafsson L, Ponten J, Zack M, Adami HO (1997). International incidence rates of invasive cervical cancer after introduction of cytological screening. Cancer Causes Control.

[2] Melnikow J, Nuovo J, Willan AR, Chan BK, Howell LP (1998). Natural history of cervical squamous intraepithelial lesions: a meta-analysis. Obstet Gynecol..

[3] Renshaw AA (2002). Measuring sensitivity in gynecologic cytology: a review. Cancer.

[4] Wentzensen N, von Knebel DM (2007). Biomarkers in cervical cancer screening. Dis Markers..

[5] Sherman ME, Solomon D, Schiffman M, Group ALTS (2001). Qualification of ASCUS. A comparison of equivocal LSIL and equivocal HSIL cervical cytology in the ASCUS LSIL Triage Study. Am J Clin Pathol.

[6] Layfield LJ, Qureshi MN (2005). HPV DNA testing in the triage of atypical squamous cells of undetermined significance (ASCUS): cost comparison of two methods. Diagn Cytopathol..

[7] Ferris DG, Wright TC, Litaker MS, Richart RM, Lorincz AT, Sun XW (1998). Triage of women with ASCUS and LSIL on Pap smear reports: management by repeat Pap smear, HPV DNA testing, or colposcopy?. J Fam Pract..

[8] Arbyn M, Buntinx F, Van Ranst M, Paraskevaidis E, Martin-Hirsch P, Dillner J (2004). Virologic versus cytologic triage of women with equivocal Pap smears: a meta-analysis of the accuracy to detect high-grade intraepithelial neoplasia. J Natl Cancer Inst..

[9] Arbyn M, Paraskevaidis E, Martin-Hirsch P, Prendiville W, Dillner J (2005). Clinical utility of HPV-DNA detection: triage of minor cervical lesions, follow-up of women treated for high-grade CIN: an update of pooled evidence. Gynecol Oncol..

[10] Petry KU, Luyten A, Scherbring S (2013). Accuracy of colposcopy management to detect CIN3 and invasive cancer in women with abnormal screening tests: results from a primary HPV screening project from 2006 to 2011 in Wolfsburg. Germany. Gynecol Oncol..

[11] Trivers KF, Benard VB, Eheman CR, Royalty JE, Ekwueme DU, Lawson HW (2009). Repeat pap testing and colposcopic biopsies in the underserved. Obstet Gynecol..

[12] Stoler MH, Schiffman M, Atypical Squamous Cells of Undetermined Significance-Low-grade Squamous Intraepithelial Lesion Triage Study G (2001). Interobserver reproducibility of cervical cytologic and histologic interpretations: realistic estimates from the ASCUS-LSIL Triage Study. JAMA..

[13] Wentzensen N, Sherman ME, Schiffman M, Wang SS (2009). Utility of methylation markers in cervical cancer early detection: appraisal of the state-of-the-science. Gynecol Oncol..

[14] Shivapurkar N, Sherman ME, Stastny V, Echebiri C, Rader JS, Nayar R (2007). Evaluation of candidate methylation markers to detect cervical neoplasia. Gynecol Oncol..

[15] Apostolidou S, Hadwin R, Burnell M, Jones A, Baff D, Pyndiah N (2009). DNA methylation analysis in liquid-based cytology for cervical cancer screening. Int J Cancer..

[16] Szalmas A, Konya J (2009). Epigenetic alterations in cervical carcinogenesis. Semin Cancer Biol..

[17] Steenbergen RD, Snijders PJ, Heideman DA, Meijer CJ (2014). Clinical implications of (epi)genetic changes in HPV-induced cervical precancerous lesions. Nat Rev Cancer..

[18] Lai HC, Lin YW, Huang TH, Yan P, Huang RL, Wang HC (2008). Identification of novel DNA methylation markers in cervical cancer. Int J Cancer..

[19] Hansel A, Steinbach D, Greinke C, Schmitz M, Eiselt J, Scheungraber C (2014). A promising DNA methylation signature for the triage of high-risk human papillomavirus DNA-positive women. PLoS One..

[20] Lendvai A, Johannes F, Grimm C, Eijsink JJ, Wardenaar R, Volders HH (2012). Genome-wide methylation profiling identifies hypermethylated biomarkers in high-grade cervical intraepithelial neoplasia. Epigenetics..

[21] Brebi P, Maldonado L, Noordhuis MG, Ili C, Leal P, Garcia P (2014). Genome-wide methylation profiling reveals Zinc finger protein 516 (ZNF516) and FK-506-binding protein 6 (FKBP6) promoters frequently methylated in cervical neoplasia, associated with HPV status and ethnicity in a Chilean population. Epigenetics..

[22] Hesselink AT, Heideman DA, Steenbergen RD, Coupe VM, Overmeer RM, Rijkaart D (2011). Combined promoter methylation analysis of CADM1 and MAL: an objective triage tool for high-risk human papillomavirus DNA-positive women. Clin Cancer Res..

[23] Eijsink JJ, Lendvai A, Deregowski V, Klip HG, Verpooten G, Dehaspe L (2012). A four-gene methylation marker panel as triage test in high-risk human papillomavirus positive patients. Int J Cancer..

[24] Eads CA, Danenberg KD, Kawakami K, Saltz LB, Blake C, Shibata D (2000). MethyLight: a high-throughput assay to measure DNA methylation. Nucleic Acids Res..

[25] Lai HC, Lin YW, Huang RL, Chung MT, Wang HC, Liao YP (2010). Quantitative DNA methylation analysis detects cervical intraepithelial neoplasms type 3 and worse. Cancer..

[26] Chao TK, Ke FY, Liao YP, Wang HC, Yu CP, Lai HC (2013). Triage of cervical cytological diagnoses of atypical squamous cells by DNA methylation of paired boxed gene 1 (PAX1). Diagn Cytopathol..

[27] Ehrich M, Nelson MR, Stanssens P, Zabeau M, Liloglou T, Xinarianos G (2005). Quantitative high-throughput analysis of DNA methylation patterns by base-specific cleavage and mass spectrometry. Proc Natl Acad Sci U S A..

[28] Claus R, Wilop S, Hielscher T, Sonnet M, Dahl E, Galm O (2012). A systematic comparison of quantitative high-resolution DNA methylation analysis and methylation-specific PCR. Epigenetics..

[29] Bullinger L, Ehrich M, Dohner K, Schlenk RF, Dohner H, Nelson MR (2009). Quantitative DNA methylation predicts survival in adult acute myeloid leukemia. Blood..

[30] Ehrich M, Field JK, Liloglou T, Xinarianos G, Oeth P, Nelson MR (2006). Cytosine methylation profiles as a molecular marker in non-small cell lung cancer. Cancer Res..

[31] Wu B, Abbott T, Fishman D, McMurray W, Mor G, Stone K (2003). Comparison of statistical methods for classification of ovarian cancer using mass spectrometry data. Bioinformatics..

[32] Akay MF (2009). Support vector machines combined with feature selection for breast cancer diagnosis. Exp Syst Appl..

[33] Noble WS (2006). What is a support vector machine?. Nat Biotech..

[34] Vapnik V (2000). The nature of statistical learning theory.

[35] Guyon I, Weston J, Barnhill S, Vapnik V (2002). Gene selection for cancer classification using support vector machines. Mach Learn..

[36] Archer KJ, Kirnes RV (2008). Empirical characterization of random forest variable importance measures. Comput Stat Data An..

[37] Yang HJ, Liu VW, Wang Y, Chan KY, Tsang PC, Khoo US (2004). Detection of hypermethylated genes in tumor and plasma of cervical cancer patients. Gynecol Oncol..

[38] Widschwendter A, Muller HM, Fiegl H, Ivarsson L, Wiedemair A, Muller-Holzner E (2004). DNA methylation in serum and tumors of cervical cancer patients. Clin Cancer Res..

[39] Lim EH, Ng SL, Li JL, Chang AR, Ng J, Ilancheran A (2010). Cervical dysplasia: assessing methylation status (Methylight) of CCNA1, DAPK1, HS3ST2, PAX1 and TFPI2 to improve diagnostic accuracy. Gynecol Oncol..

[40] Lim SP, Wong NC, Suetani RJ, Ho K, Ng JL, Neilsen PM (2012). Specific-site methylation of tumour suppressor ANKRD11 in breast cancer. Eur J Cancer..

[41] Hammons GJ, Yan-Sanders Y, Jin B, Blann E, Kadlubar FF, Lyn-Cook BD (2001). Specific site methylation in the 5'-flanking region of CYP1A2 interindividual differences in human livers. Life Sci..

[42] Song SH, Jong HS, Choi HH, Kang SH, Ryu MH, Kim NK (2000). Methylation of specific CpG sites in the promoter region could significantly down-regulate p16(INK4a) expression in gastric adenocarcinoma. Int J Cancer..

[43] Nile CJ, Read RC, Akil M, Duff GW, Wilson AG (2008). Methylation status of a single CpG site in the IL6 promoter is related to IL6 messenger RNA levels and rheumatoid arthritis. Arthritis Rheum..

[44] Roberson ED, Liu Y, Ryan C, Joyce CE, Duan S, Cao L (2012). A subset of methylated CpG sites differentiate psoriatic from normal skin. J Invest Dermatol..

[45] Claus R, Lucas DM, Stilgenbauer S, Ruppert AS, Yu L, Zucknick M (2012). Quantitative DNA methylation analysis identifies a single CpG dinucleotide important for ZAP-70 expression and predictive of prognosis in chronic lymphocytic leukemia. J Clin Oncol..

[46] Sohn BH, Park IY, Lee JJ, Yang SJ, Jang YJ, Park KC (2010). Functional switching of TGF-beta1 signaling in liver cancer via epigenetic modulation of a single CpG site in TTP promoter. Gastroenterology..

[47] Peille AL, Brouste V, Kauffmann A, Lagarde P, Le Morvan V, Coindre JM (2013). Prognostic value of PLAGL1-specific CpG site methylation in soft-tissue sarcomas. PLoS One..

[48] Yuan J, Luo RZ, Fujii S, Wang L, Hu W, Andreeff M (2003). Aberrant methylation and silencing of ARHI, an imprinted tumor suppressor gene in which the function is lost in breast cancers. Cancer Res..

[49] Enokida H, Shiina H, Urakami S, Igawa M, Ogishima T, Li LC (2005). Multigene methylation analysis for detection and staging of prostate cancer. Clin Cancer Res..

[50] Chih-Wei Hsu C-CC, Lin C-J. A practical guide to support vector classification. 2010. http://www.csie.ntu.edu.tw/~cjlin/papers/guide/guide.pdf. Accessed 10 Jan 2015.

[51] Hawkins DM (2004). The problem of overfitting. J Chem Inf Comput Sci..

[52] Brown MPS, Grundy WN, Lin D, Cristianini N, Sugnet CW, Furey TS (2000). Knowledge-based analysis of microarray gene expression data by using support vector machines. Proc Natl Acad Sci..

[53] Vapnik V (1998). The nature of statistical learning theory.

[54] Yang ZR (2004). Biological applications of support vector machines. Brief Bioinform..

[55] Efron B, Gong G (1983). A leisurely look at the bootstrap, the jackknife, and cross-validation. Am Stat..

[56] Ben-Hur A, Weston J (2010). A user’s guide to support vector machines. Methods Mol Biol..

[57] Fu WJ, Carroll RJ, Wang S (2005). Estimating misclassification error with small samples via bootstrap cross-validation. Bioinformatics..

[58] Overmeer RM, Louwers JA, Meijer CJLM, van Kemenade FJ, Hesselink AT, Daalmeijer NF (2011). Combined CADM1 and MAL promoter methylation analysis to detect (pre-)malignant cervical lesions in high-risk HPV-positive women. Int J Cancer..

[59] Galgano MT, Castle PE, Atkins KA, Brix WK, Nassau SR, Stoler MH (2010). Using biomarkers as objective standards in the diagnosis of cervical biopsies. Am J Surg Pathol..

[60] Pikor LA, Enfield KS, Cameron H, Lam WL. DNA extraction from paraffin embedded material for genetic and epigenetic analyses. J Vis Exp. 2011; 49:e2763. doi:10.3791/2763. 10.3791/2763 PMC3197328 21490570

[61] Baldi P, Brunak S, Chauvin Y, Andersen CA, Nielsen H (2000). Assessing the accuracy of prediction algorithms for classification: an overview. Bioinformatics..

