# Supplementary material for: Quantitative survey of multiple CpGs from 5 genes identifies CpG methylation panel discriminating between high- and low-grade cervical intraepithelial neoplasia
Source: Clin Epigenetics. 2015 Jan 22;7(1):4. doi: 10.1186/s13148-014-0037-1 (PMC4334603; doi:10.1186/s13148-014-0037-1)
Supplement: Additional file 1: Table S1. — Primers, respective target sizes, and locations of the CGIs analyzed by EpiTYPER. L indicates left primer; R indicates right primer. [file 13148_2014_37_MOESM1_ESM.doc]

**Additional file 1: Table S1 Primers, respective target sizes, and locations of the CGIs analyzed by EpiTYPER**

| Name | Primers | Target size (bp) | Chromosomal location of amplicons  analyzed by EpiTYPER |
| --- | --- | --- | --- |
| *LMX1A* | L:5’-TTTTAGTGATTGGAGTAGAGAGAAGTTG-3’  R:5’-TACCTTCCCTCCTAACCTTAAAAAA-3’ | 283 | chr1:  165325609-165325892 |
| *SOX1* | L:5’-GGGTATTTGGGATTAGTATATGTTTA-3’ R:5’-CACAAACCACTTACCAAAAAAA-3’ | 301 | chr13:  112721449-112721750 |
| *ONECUT1* | L:5’-TTGGTTAGTTTGAGTTATGGTTTTG-3’ R:5’-CTAAACCCCCTCCTTCATTTATACC-3’ | 197 | chr15:  53082501-53082698 |
| *NKX6.1* | L:5’-GTTGTTGGATTTGTGTTTTTTTA-3’ R:5’-CCCTCAAATCTAATTCCAAAACC-3’ | 214 | chr4: 85414496-85414710 |
| *PAX1* | L:5’-GATTTAGTTTTGGGTTTTGGAGAAG-3’ R:5’-TTCCAAAAATAACCTATAAATCCCC-3’ | 247 | chr20: 21686729-21686976 |

L indicates left primer; R indicates right primer.
